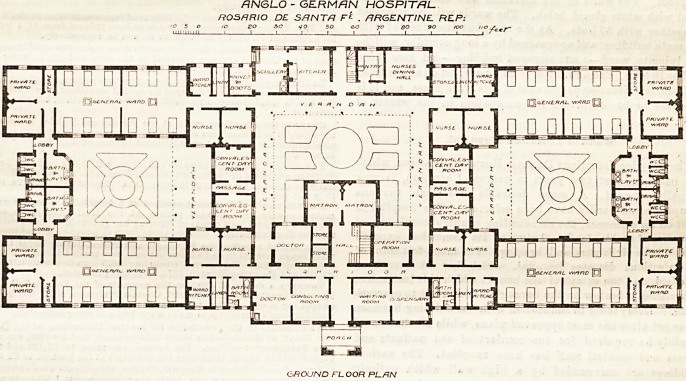# Hospital Construction

**Published:** 1896-08-15

**Authors:** 


### HOSPITAL CONSTRUCTION.

### ANGLO-GERMAN HOSPITAL, ROSARIO DE SANTA FE, ARGENTINE REPUBLIC.

The construction of hospitals in other countries than our own may be a matter of interest to many of our readers, and we publish herewith the plans of a hospital built in 1890 and 1891 by subscription among the English and German residents of Rosario, and supported by their voluntary contributions. The plans are the work of Messrs. R. and E. Conder, who seem to have had many difficulties to contend with both in local materials and local craftsmen. A portion only of the design here shown, viz., two large wards, the administrative and service blocks, has been carried out. The walls are of local brick, plastered externally with cement, and the roofs are of corrugated iron laid on boarding—the latter covered with felt. Two air spaces are thus provided—one between the iron and felt and the other between the boarding and ceilings of the rooms—and these have been found very satisfactory in securing a comparatively even temperature both in summer and winter.

The complete plans represent buildings formed round three courtyards connected by passages. The wards and their adjuncts surrounding the two extreme courts, and the administrative and kitchen blocks, joining these buildings together, form the sides of central courtyard.

The general entrance is in the centre of the building. On the left hand is a consulting room and doctor's room, connected together, and on the right a waiting-room and dispensary, also connected. A staircase opposite the entrance leads to rooms on the upper floor of which we have neither plan nor description, and two rooms assigned to the matron lie beyond this staircase. From the entrance hall corridors run right and left to the wards. Out of that to the right and opposite the dispensary the operation room opens, and a second "doctor's room" is shown in the corresponding position on the left. The buildings surrounding the two side courts are precisely similar in arrangement, and consist in each case of two wards for twelve beds, lying parallel to one another on opposite sides of the court, and separated by a passage from two private wards projecting beyond the court. The sanitary appliances for the wards lie across one end of the court, and nurses' rooms and convalescent rooms across the other. The convalescent rooms open out of passages connecting the central and side courts, and are lighted by windows looking on verandahs.

The corridors giving access to the wards have ward kitchens and linen rooms on one side, and nurses' rooms on the other.

The heating of the wards is by central stoves. The beds are ranged on both sides of the wards, but no window is provided between the end bed and the wall. A cross-ventilated lobby properly cuts off the sanitary block from the wards, but a sluice would probably be practically more useful than the urinals shown. The plans do not show how any control can be exercised over the wards from the ward-kitchens or nurses' rooms, and the private wards are badly placed in this respect.

The arrangement of w.c.'s accessible through bath-rooms (though presumably for the use of the medical men and nurses only) is not good, and the nurses' rooms lighted from covered passages are an obvious defect. The matron's rooms do not seem rightly

ANGLO-GERMAN HOSPITAL
ROSARIO DE SANTA F$^{E}$. ARGENTINE REP:

GROUND FLOOR PLAN

placed either with regard to the kitchen department or the entrance. The operation room is awkward of access for many of the ward patients, and its light appears inadequate. The connecting door between the waiting-room and dispensary is unusual and questionable. The verandahs are no doubt dictated by the climate, but the want of direct light to some of the passages and corridors is a blot. The convalescent rooms, though small, are, good points in the plans. Possibly some of these details may be reconsidered before the whole scheme is completed.

## Figures and Tables

**Figure f1:**